# Comparison of two experimental ARDS models in pigs using electrical impedance tomography

**DOI:** 10.1371/journal.pone.0225218

**Published:** 2019-11-13

**Authors:** Nadine Hochhausen, Jakob Orschulik, Andreas Follmann, Susana Aguiar Santos, Henriette Dohmeier, Steffen Leonhardt, Rolf Rossaint, Michael Czaplik

**Affiliations:** 1 Department of Anesthesiology, Medical Faculty, RWTH Aachen University, Aachen, Germany; 2 Philips Chair for Medical Information Technology, RWTH Aachen University, Aachen, Germany; National Yang-Ming University, TAIWAN

## Abstract

**Background:**

Animal trials contribute to major achievements in medical science. The so-called lavage model is frequently used to evaluate ventilation strategies in acute respiratory distress syndrome (ARDS) using electrical impedance tomography (EIT). But, the lavage model itself might have systematic impacts on EIT parameters. Therefore, we established an additional experimental model, in which ARDS is caused by intravenously administered lipopolysaccharide (LPS). In this study, we want to examine if EIT measurements provide consistent results in both experimental models or whether the pathophysiology of the model influences the findings. Overall, we want to compare both experimental models regarding clinical parameters and EIT-derived indices, namely the global inhomogeneity (GI) index and the regional ventilation delay (RVD) index.

**Methods:**

Nineteen pigs were included in this study, allocated to the control group (CO; n = 5), lavage group (LAV; n = 7) and LPS group (LPS; n = 7). After baseline measurements and the establishment of ARDS, assessment of respiratory mechanics, hemodynamics, gas exchange and EIT recordings were performed hourly over eight hours.

**Results:**

In both experimental ARDS models, EIT measurements provided reliable results. But, the GI and the RVD index did not show consistent results as compared to the CO group. Initially, GI and RVD index were higher in the LAV group but not in the LPS group as compared to the CO group. This effect disappeared during the study. Furthermore, the GI index and the RVD index were higher in the LAV group compared to the LPS group in the beginning as well. This, once again, disappeared. Clinical lung injury parameters remained more stable when using LPS.

**Conclusion:**

The two models showed quite different influences on the GI and RVD index. This implies, that the underlying pathophysiology affects EIT parameters and thus the findings. Hence, translation to EIT-guided clinical therapy in humans suffering from ARDS might be limited.

## Introduction

Animal models are crucial to the study of therapies for serious diseases, such as acute respiratory distress syndrome (ARDS). ARDS is a life-threatening condition with a high mortality, even though the clinical syndrome was described more than 50 years ago [1,2]. ARDS can be caused by a variety of pulmonary (e.g., aspiration, pneumonia) or non-pulmonary (e.g., sepsis, trauma) diseases [3,4]. The final common path is hypoxemia, decreased lung compliance, edema and bilateral infiltrates on chest radiographs. Despite decades of intense research, up-to-date therapeutic options remain limited. Animal trials help to develop and to evaluate new therapies.

A common established model of ARDS in large animals is the lavage model [5,6]. The mechanism of lung injury is based on surfactant depletion leading to an increase in alveolar surface tension with consecutive alveolar collapse, decreased lung compliance, edema and, finally, an impaired gas exchange. The results are similar to clinical ARDS but do not represent the usual course of pathophysiological changes. First, the altered gas exchange normally improves in contrast to the clinical course within hours during the study [5,7]. Second, the inflammatory response does not reflect ARDS accurately [5] and, third, saline solution may remain in the lungs after the repetitive lavages, leading to arbitrary effects. Despite these systematic weaknesses, it is frequently used in combination with electrical impedance tomography (EIT) [8,9].

EIT is a radiation-free, non-invasive bedside-available technique that enables to visualize ventilation by measuring time-dependent electrical impedance variations. Relatively sophisticated reconstruction algorithms lead to images and video sequences, respectively [10,11]. EIT-derived indices were developed to simplify interpretation and to facilitate monitoring [12–15]. However, the above mentioned limitations might affect the validity of the global inhomogeneity (GI) index and the regional ventilation delay (RVD) index.

For this reason, we established a sepsis-induced ARDS model using lipopolysaccharide (LPS), which is closer to the pathophysiology of clinical ARDS. LPS is commonly localized in the outer membrane of gram-negative bacteria, e.g., Escherichia coli. To induce an experimental ARDS, LPS must be either infused intravenously or administered intratracheally. The mechanism of LPS-induced ARDS is based on damage to endothelial cells and a systemic inflammatory response. Therefore, the LPS-induced ARDS model imitates the pathophysiology of clinical sepsis, which is one of the most prevalent causes of ARDS in humans [5,7,16].

In this study, we want to investigate if EIT measurements provide consistent results in both ARDS models, namely the LAV and the LPS model, or whether the pathophysiology of the model influences the findings. Additionally, we want to compare both experimental models regarding clinical parameters and EIT-derived indices, namely the GI index and the RVD index.

## Materials and methods

After study approval, provided by the North Rhine-Westphalia State Agency for Nature, Environment, and Consumer Protection (Germany; 84–02.04.2013.A200), the study was performed in laboratories at the University Hospital Aachen. All animals were housed for seven days before entering the study and were thus acclimatized to their new surroundings. The animals were kept in groups on litter and had free access to water. The experiments were performed according to the Guidance for the Care and Use of Laboratory Animals [17].

### Animal preparation

Nineteen juvenile female pigs (German Landrace), weighing on average 36.8 ± 2.5 kg (mean±SD), were included in this study. Before the experiments began, the health status of each animal was assessed by a veterinarian.

In the mornings, intramuscular (i.m.) premedication with 1 ml 1% atropine, 0.2 ml/kg azaperone and 1 ml 10% ketamine, followed by an intravenous (i.v.) access (ear vein), was performed. Thereafter, anesthesia induction with approximately 2.5–4.5 mg/kg pentobarbital via ear vein was carried out, followed by endotracheal intubation. Then, a urinary catheter was inserted by natural route. During the study, the animals were placed in the supine position on a heated blanket to maintain normothermic body temperature. Anesthesia was maintained intravenously with fentanyl (3–10 μg/kg/h) and thiopental (4–10 mg/kg/h). The depth of anesthesia was controlled by blood pressure and heart rate according to human clinical standards. Moreover, a balanced crystalloid solution (Ringer; 0.1 ml/kg/min) was infused continuously for adequate fluid replacement, and mean arterial pressure (MAP) was stabilized with additional balanced crystalloid infusion and catecholamine therapy, if required. All animals were ventilated mechanically in a volume-controlled mode using a tidal volume of 6 (to 8)ml/kg bodyweight, an inspiratory–expiratory ratio of 1:1, a fraction of inspired oxygen (F_i_O_2_) of 0.3 and a positive end- expiratory pressure (PEEP) of 5 cm H_2_O. Breathing rate was adjusted to maintain a partial pressure of carbon dioxide (p_a_CO_2_) of 35–45 mmHg. After placing the femoral arterial and the central venous catheter using the Seldinger technique as well as the pulmonary artery catheter for cardiac output measurements, the necessary preparations were completed. All animals were connected to a Datex monitor (Datex AS3 Monitoring System, Datex-Ohmeda) for continuous vital data assessment (hemodynamic parameters and respiratory parameters, including the calculated Horowitz index, defined as arterial oxygen pressure (P_a_O_2_) divided by F_i_O_2_). All blood gas measurements were analyzed by a blood gas analyzer (ABL800 blood gas analyzer, Radiometer, Copenhagen, Denmark).

### Experimental protocol

The lavage group (LAV group), the LPS group (LPS group) and the control group (CO group) were studied successively. After initial preparations, baseline measurements were performed. Thereafter, F_i_O_2_ was set to 1.0, and lung injury was induced in both experimental groups either with repetitive lavages or with LPS.

In the LAV group, lavages with 30 ml/kg bodyweight saline solution 0.9% were repeated every 10 min, followed by blood gas analysis. When P_a_O_2_ was below 100 mmHg one hour after the last lavage (in general, after three hours of repeated lavages), ARDS was defined to be established. One hour later (ARDS 1), the criteria for severe (P_a_O2/FiO_2_ < 100 mmHg) to moderate ARDS (P_a_O_2_/F_i_O_2_ between 100 and 200 mmHg) were fulfilled. Unfortunately, two animals of the LAV group showed criterias of a mild ARDS (P_a_O_2_/F_i_O_2_ between 200 and 300 mmHg). In the LPS group, 200 μg/kg LPS (Escherichia coli, Sigma 055:B5) was infused for one hour. Three hours after LPS infusion, the criteria for severe (P_a_O2/FiO_2_ < 100 mmHg) to moderate ARDS (P_a_O_2_/F_i_O_2_ between 100 and 200 mmHg) were fulfilled (ARDS 1). In the CO group, all animals were ventilated with a F_i_O_2_ of 1.0 after baseline measurements for three hours as well.

In all groups, ventilation parameters, hemodynamic measurements, blood gas analysis and EIT recordings were collected at baseline measurement and hourly up to eight hours after establishment of ARDS. In the CO group, measurements were performed hourly in accordance with both experimental groups.

### EIT measurements and analysis

For EIT monitoring, a 16-electrode belt was fastened around the thorax at the 5^th^ intercostal space. The belt was connected to the PulmoVista500 (Draeger Medical, Luebeck, Germany), and an EIT sequence of two minutes was recorded with a sampling rate of 50 Hz at each predefined time point (at baseline measurement and hourly after establishment of ARDS). For offline analysis, Data Review 5.0 software (Draeger Medical, Luebeck, Germany) was used for image reconstruction, and the EIT Diag v1.6 software (Drager Medical GmbH, Luebeck, Germany) was subsequently used for calculating GI index and RVD index. For reconstruction, we chose a ROI-threshold of 0.30 in the recordings settings. Then, EIT recordings of all four time points (Baseline, ARDS 1, ARDS 4, ARDS 8) were imported separately for each animal. EIT images were reconstructed after selecting individual sequences. Hereafter, EIT images were post-processed manually if artefacts in the reconstructed lung were visible.

The GI index was first described by Zhao et al. [18,19]. It represents heterogeneity of the lung. The higher the value, the worse the disorder in ventilation distribution.

$$GI=\frac{\sum_{x,y\in lung}\left|{DI}_{xy}-Median({DI}_{lung})\right|}{\sum_{x,y\in lung}{DI}_{xy}}$$

DI = “differential impedance,” DIxy = pixels in a defined lung region,

DI_lung_ = All pixels representing the lung

The RVD index represents regional ventilation distribution and characterizes the percentage of time needed to achieve a threshold of 40% of the regional impedance change (compared to the total inspiratory time) [15]. The RVD index represents the inhomogeneity of the lung and is well correlated with the amount of tidal recruitment raised by computed tomography.

$$RVD40=\frac{\Delta t40\%}{tmax-tmin}*100\%$$

RVD = Regional Ventilation Delay index; Δt40% = percentage of time needed to achieve a threshold of 40% of the regional impedance change;

tmax-tmin = inflation time

### Statistical methods

#### No power analysis was performed in this study

All data were analyzed with SPSS Statistics 23 for Windows (SPSS Inc., IBM Business Analytics Software, Armonk, NY, USA). Not normally distribution was confirmed using the Shapiro-Wilk test. Therefore, median values and interquartile ranges were presented. The Mann-Whitney-U test was used to analyze the difference between two independent groups (LPS and CO groups; LAV and CO groups; LAV and LPS groups). The statistical significance level was set at p<0.05. Due to the fact, that there are four time points, we performed a multiple comparison analysis according to a Bonferroni correction for RVD index and GI index in addition. Hereafter, the significance level must be considered at p<0.0125.

## Results

In total, nineteen animals were examined in this study. Five animals served as controls, seven animals were studied in the LAV group, and seven animals were studied in the LPS group. Three animals (two animals of the LAV group and one animal of the LPS group) died because of severe impairment of gas exchange due to severe ARDS before completing the study protocol. The data of these animals are included until the last useful measurement (LPS animal: two hours after ARDS; both LAV animals: three hours after ARDS).

The baseline data of all animals showed no differences except for systolic pulmonary arterial pressure (SPAP). However, SPAP was significantly higher in the CO group compared to the LAV group (Table 1).

**Table 1:** Baseline values of gas exchange, EIT- indices, respiratory mechanics and hemodynamics.

	LPS group	LAV group	CO group	p-value (LPS vs LAV)	p-value (LPS vs CO)	p-value (LAV vs CO)
**GI index**	26.8 (25.8–28.3)	26.2 (24.7–28.9)	26.9 (25.3–27.6)	0.730	0.841	0.730
**RVD index**	2.8 (2.6–5.7)	3.1 (2.9–5.0)	4.0 (3.8–4.7)	0.730	0.730	0.343
**Compliance, ml/mbar**	19.4 (17.9–21.7)	20.4 (19.0–23.2)	19.3 (17.4–19.5)	0.209	0.432	0.073
**PIP, mbar**	20 (19–21)	18 (17–20)	19 (18–20)	0.097	0.268	0.432
**RR, /min**	32 (32–34)	30 (28–30)	30 (28–33)	0.383	0.268	0.639
**Horowitz Index**	510 (435–530)	524 (477–584)	525 (497–551)	0.535	0.343	0.935
**SAP, mmHg**	103 (99–109)	109 (102–115)	106 (102–129)	0.318	0.268	0.755
**SPAP, mmHg**	26 (23–28)	24 (16–25)	30 (27–31)	0.181	0.177	0.010*
**HR, /min**	124 (107–154)	113 (104–136)	126 (122–150)	0.383	0.639	0.202
**Cardiac Output, l/min**	4.3 (3.7–5.1)	4.7 (4.6–5.2)	4.9 (4.7–5.8)	0.534	0.177	0.432
**Lactate, mmol**	0.9 (0.4–3.8)	1.1 (0.9–3.3)	1.9 (1.0–2.2)	0.805	0.876	0.755
**Total balanced crystalloid solution, ml**	900 (850–1400)	1000 (1000–1400)	1000 (1000–1100)	0.209	0.432	0.755
**Total urine output, ml**	100 (50–200)	50 (0–600)	100 (50–150)	1.00	0.876	1.00

Abbreviations: GI index = global inhomogeneity index, RVD index = regional ventilation delay index, PIP = Peak Inspiratory Pressure, RR = Respiratory Rate; SAP = Systolic arterial pressure; SPAP = Systolic pulmonary arterial pressure; HR = Heart rate; LPS group = Lipopolysaccharide group; LAV group = Lavage group; CO group = Control group

* = p<0.05

### LPS group and LAV group versus CO group

**Fig 1:**
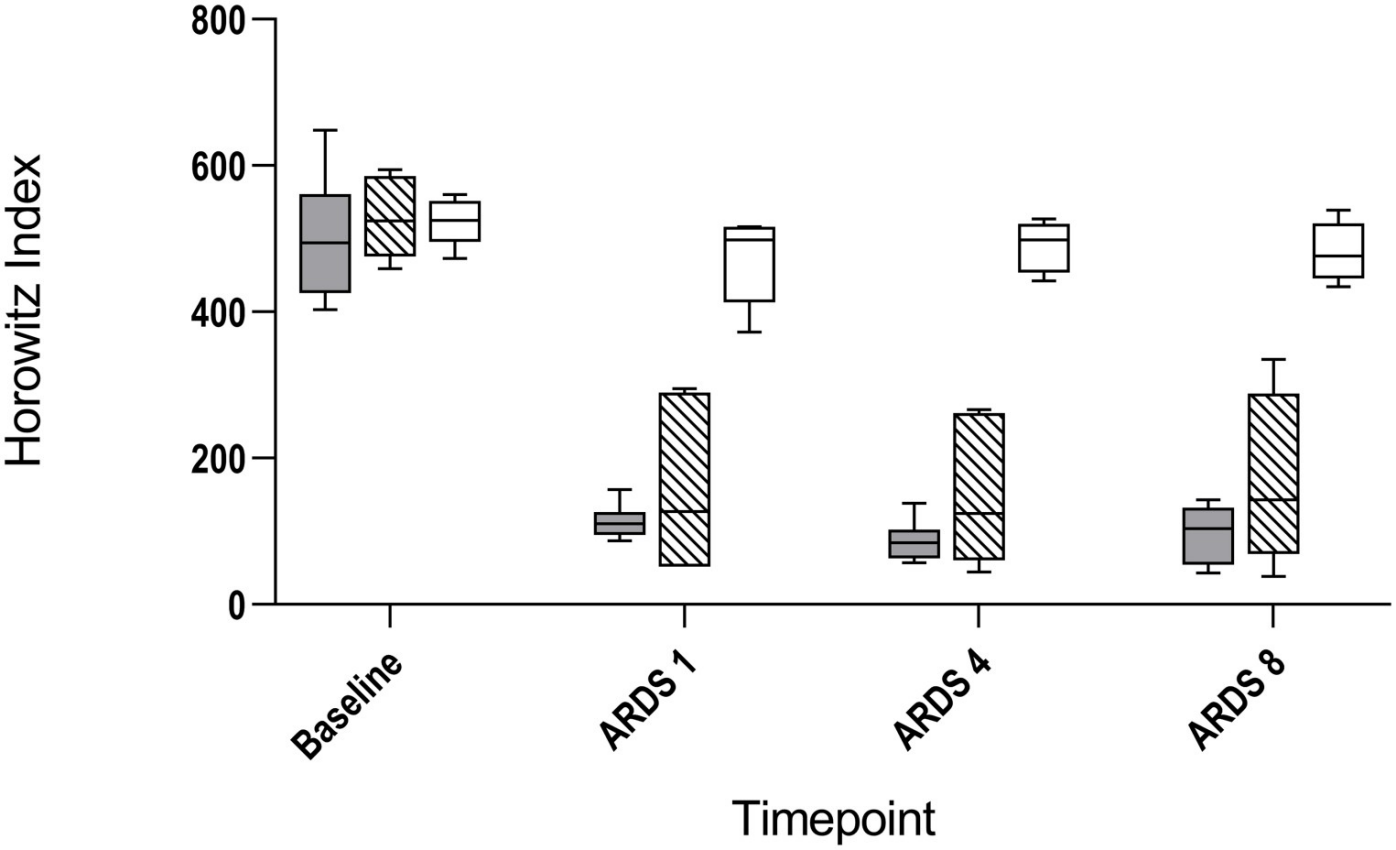
Course of Horowitz index of lavage induced lung injury, lipopolysaccharide induced lung injury and control group. Changes of Horowitz index for lipopolysaccharide induced lung injury (LPS; grey), lavage induced lung injury (LAV; stripes) and control group (CO; white) at baseline, one hour (ARDS 1), four hours (ARDS 4) and eight hours (ARDS 8) after ARDS.

ARDS was successfully induced in all LPS and LAV group animals (Fig 1).

Gas exchange and respiratory mechanics differed significantly compared to the CO group. Therefore, one hour (ARDS 1), four hours (ARDS 4) and eight hours (ARDS 8) after ARDS, Horowitz index and compliance were significantly lower in both experimental groups as compared to the CO group, whereas respiratory rate (RR) was higher in LPS group during the whole study. The LAV group showed a higher RR only at time point ARDS 1 (Tables 2–4, Fig 1).

**Table 2:** ARDS 1 values of gas exchange, EIT- indices, respiratory mechanics and hemodynamics.

	LPS group	LAV group	CO group	p-value (LPS vs LAV)	p-value (LPS vs CO)	p-value (LAV vs CO)
**GI index**	25.4 (24.5–26.4)	27.3 (26.5–28.8)	26.4 (25.3–26.9)	0.026*	0.329	0.082
**RVD index**	3.1 (3.0–4.2)	4.6 (4.3–6.3)	3.5 (3.1–3.9)	0.026*	0.792	0.004*^b^
**Compliance, ml/mbar**	12.0 (9.2–13.2)	8.7 (6.7–12.2)	18.1 (17.0–19.3)	0.038*	0.003*	0.003*
**PIP, mbar**	27 (26–32)	37 (30–38)	20 (19–21)	0.017*	0.003*	0.003*
**RR, /min**	35 (32–38)	36 (28–38)	25 (24–28)	0.805	0.003*	0.030*
**Horowitz Index**	110 (96–125)	127 (53–288)	498 (415–515)	0.805	0.003*	0.003*
**SAP, mmHg**	110 (103–119)	120 (116–127)	147 (120–149)	0.038*	0.010*	0.106
**SPAP, mmHg**	56 (54–57)	44 (26–54)	29 (28–34)	0.051	0.004*	0.003*
**HR, /min**	157 (142–171)	84 (77–129)	81 (77–99)	0.002*	0.003*	0.432
**Cardiac output, l/min**	3.7 (3.1–5.0)	3.7 (3.4–4.3)	3.5 (2.9–3.8)	0.731	0.429	0.268
**Lactate, mmol**	2.7 (2.4–3.4)	0.7 (0.6–0.7)	0.5 (0.4–0.7)	0.001*	0.003*	0.073
**Total balanced crystalloid solution, ml**	2900 (2400–3300)	2400 (2000–2500)	2400 (2200–2900)	0.128	0.343	0.755
**Total urine output, ml**	400 (250–500)	800 (500–1300)	650 (400–850)	0.026*	0.073	0.268

Abbreviations: GI index = global inhomogeneity index, RVD index = regional ventilation delay index, PIP = Peak Inspiratory Pressure, RR = Respiratory Rate; SAP = Systolic arterial pressure; SPAP = Systolic pulmonary arterial pressure; HR = Heart Rate; LPS group = Lipopolysaccharide group; LAV group = Lavage group; CO group = Control group, ARDS 1 = One hour after ARDS

* = p<0.05;

^b^ = p<0.0125 according to Bonferroni correction

**Table 3:** ARDS 4 values of gas exchange, EIT- indices, respiratory mechanics and hemodynamics.

	LPS group	LAV group	CO group	p-value (LPS vs LAV)	p-value (LPS vs CO)	p-value (LAV vs CO)
**GI index**	26.8 (26.1–27.2)	28.4 (26.9–29.7)	26.0 (25.4–26.6)	0.067	0.247	0.032*
**RVD index**	5.2 (3.9–6.7)	7.4 (5.3–7.6)	4.0 (2.9–4.6)	0.114	0.126	0.032*
**Compliance, ml/mbar**	11.1 (10.3–12.3)	7.6 (6.7–12.2)	17.9 (16.5–18.7)	0.429	0.004*	0.008*
**PIP, mbar**	28 (26–30)	36 (29–39)	20 (20–21)	0.052	0.004*	0.008*
**RR, /min**	34 (32–35)	28 (27–38)	25 (24–28)	0.662	0.004*	0.095
**Horowitz Index**	84 (64–101)	124 (61–261)	498 (455–519)	0.537	0.004*	0.008*
**SAP, mmHg**	109 (93–118)	132 (124–139)	116 (115–129)	0.017*	0.126	0.056
**SPAP, mmHg**	46 (41–53)	44 (39–55)	31 (27–37)	0.690	0.008*	0.016*
**HR, /min**	171 (144–208)	93 (70–120)	75 (72–101)	0.009*	0.009*	0.690
**Cardiac output, l/min**	3.0 (2.3–3.6)	3.3 (2.9–3.7)	3.0 (2.9–3.4)	0.548	0.917	0.548
**Lactate, mmol**	1.8 (1.5–2.5)	0.5 (0.45–0.6)	0.4 (0.4–0.6)	0.004*	0.004*	0.310
**Total balanced crystalloid solution, ml**	3600 (3275–4800)	3100 (2850–3625)	3100 (2800–3500)	0.052	0.082	1.00
**Total urine output, ml**	500 (375–638)	1300 (925–2900)	900 (600–1250)	0.009*	0.030*	0.151

Abbreviations: GI index = global inhomogeneity index, RVD index = regional ventilation delay index, PIP = Peak Inspiratory Pressure; RR = Respiratory Rate; SAP = Systolic arterial pressure; SPAP = Systolic pulmonary arterial pressure; HR = Heart Rate; LPS group = Lipopolysaccharide group; LAV group = Lavage group; CO group = Control group, ARDS 4 = Four hours after ARDS

* = p<0.05

**Table 4:** ARDS 8 values of gas exchange, EIT- indices, respiratory mechanics and hemodynamics.

	LPS group	LAV group	CO group	p-value (LPS vs LAV)	p-value (LPS vs CO)	p-value (LAV vs CO)
**GI index**	25.5 (25.1–28.4)	27.8 (25.7–29.1)	26.6 (25.5–27.1)	0.421	0.690	0.421
**RVD index**	5.3 (3.3–6.2)	6.9 (5.4–11.2)	4.3 (3.6–5.8)	0.082	0.792	0.056
**Compliance, ml/mbar**	12.4 (10.7–13.4)	7.4 (6.9–11.8)	17.6 (16.1–17.9)	0.052	0.004*	0.008*
**PIP, mbar**	27 (26–29)	38 (29–39)	21 (20–22)	0.017*	0.004*	0.008*
**RR, /min**	35 (32–35)	38 (27–38)	25 (24–28)	0.662	0.004*	0.056
**Horowitz Index**	104 (56–131)	143 (70–287)	476 (447–519)	0.329	0.004*	0.008*
**SAP, mmHg**	99 (88–109)	114 (110–128)	126 (116–135)	0.017*	0.004*	0.222
**SPAP, mmHg**	36 (34–42)	41 (39–52)	32 (30–34)	0.151	0.032*	0.008*
**HR, /min**	193 (154–202)	90 (80–138)	78 (76–111)	0.017*	0.004*	0.421
**Cardiac output, l/min**	4.2 (2.8–5.0)	3.4 (2.9–3.9)	3.3 (3.0–3.8)	0.421	0.548	0.917
**Lactate, mmol**	1.6 (1.5–3.6)	0.4 (0.3–0.6)	0.5 (0.5–0.6)	0.004*	0.004*	0.310
**Total balanced crystalloid solution, ml**	5300 (4563–6325)	4100 (3825–4600)	3900 (3600–4350)	0.052	0.017*	0.548
**Total urine output, ml**	550 (450–838)	2000 (1300–3400)	1150 (900–1600)	0.004*	0.009*	0.095

Abbreviations: GI index = global inhomogeneity index, RVD index = regional ventilation delay index, PIP = Peak Inspiratory Pressure; RR = Respiratory Rate; SAP = Systolic arterial pressure; SPAP = Systolic pulmonary arterial pressure; HR = Heart Rate; LPS group = Lipopolysaccharide group; LAV group = Lavage group; CO group = Control group, ARDS 8 = Eight hours after ARDS

* = p<0.05

**Fig 2:**
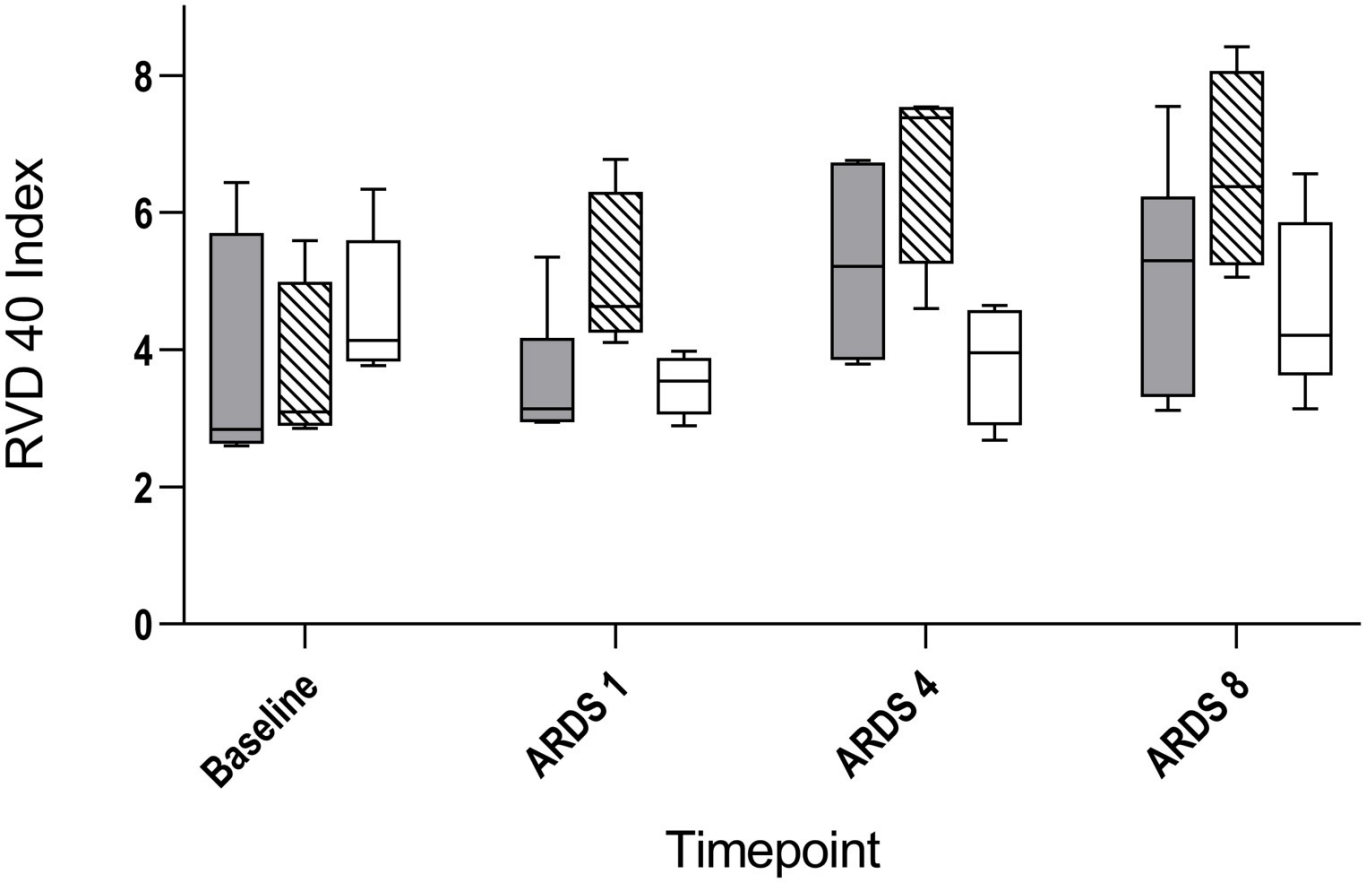
Course of regional ventilation delay index of lavage induced lung injury, lipopolysaccharide induced lung injury and control group. Changes of regional ventilation delay index (RVD index) for lipopolysaccharide induced lung injury (LPS; grey), lavage induced lung injury (LAV; stripes) and control group (CO; white) at baseline, one hour (ARDS 1), four hours (ARDS 4) and eight hours (ARDS 8) after ARDS.

**Fig 3:**
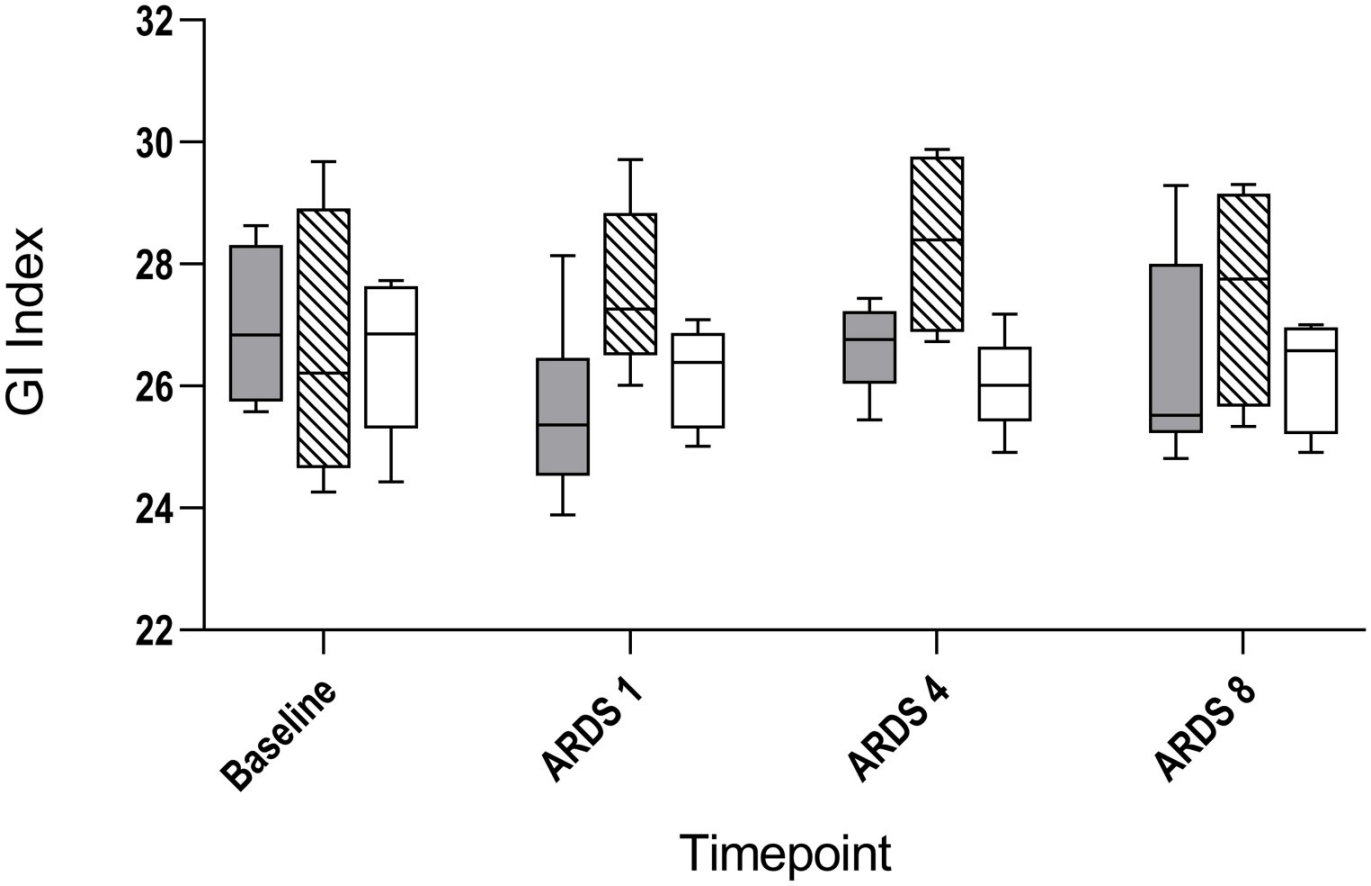
Course of global inhomogeneity index of lavage induced lung injury, lipopolysaccharide induced lung injury and control group. Changes of global inhomogeneity index (GI index) for lipopolysaccharide induced lung injury (LPS; grey), lavage induced lung injury (LAV; stripes) and control group (CO; white) at baseline, one hour (ARDS 1), four hours (ARDS 4) and eight hours (ARDS 8) after ARDS.

Furthermore, peak inspiratory pressure (PIP) and SPAP were higher in the LPS and LAV groups as compared to the CO group (Tables 2–4). Cardiac output did not differ in both experimental groups compared to CO group at any time (Tables 2–4). So far, no sufficient differences between the two experimental ARDS models were observable. However, only the LPS group demonstrated a significantly lower systolic arterial pressure (SAP) one hour and eight hours after ARDS (ARDS 1: 110 (103–119) vs. 147 (120–149); p = 0.010; ARDS 8: 99 (88–109) vs. 126 (116–135); p = 0.004), as well as a higher heart rate (HR) (Tables 1–4), despite additional balanced crystalloid infusion compared to the CO group in the later phase of ARDS (ARDS 8: 5300 (4563–6325) vs. 3900 (3600–4350); p = 0.017) and catecholamine therapy (all animals of the LPS group vs. one animal of the LAV group vs. no animal of the CO group) for hemodynamic stabilization. Furthermore, a more differentiated volume therapy was initiated in the LPS group. Five of seven LPS animals needed between 500 and 1000 ml additional volume whereas only one LAV animal needed 500 ml additional volume to maintain blood pressure. Nevertheless, total urinary output was less since ARDS 4 and lactate was higher at all times in the LPS group (Tables 1–4). Additionally, no differences in the GI and RVD indexes were observed in the LPS group as compared to the CO group (Tables 2–4). In the LAV group compared to CO group, one hour and four hours after ARDS, the RVD index was higher (ARDS 1: 4.6 (4.3–6.3) vs. 3.5 (3.1–3.9), p = 0.004; ARDS 4: 7.4 (5.3–7.6) vs 4.0 (2.9–4.6), p = 0.032; Fig 2), whereas the GI index was higher after some delay (ARDS 4: 28.4 (26.9–29.7) vs. 26.0 (25.4–26.6), p = 0.032) (Tables 2 and 3, Fig 3). However, after Bonferroni correction, only the RVD index at time point ARDS 1 demonstrated a significance between LAV and CO group (p = 0.004).

After eight hours of ARDS, no differences in EIT-derived indices were still observable between the LAV and CO groups (Table 4).

### Comparison of experimental models

Before induction of ARDS, there were no differences in the baseline values of gas exchange, respiratory mechanics, hemodynamics and EIT-derived indices between the groups (Table 1).

However, after initiation of ARDS, differences between the LPS and LAV groups were observable. In particular, the GI index and the RVD index were higher in the LAV group compared to the LPS group (GI index: ARDS 1: 25.4 (24.5–26.4) vs. 27.3 (26.5–28.8), p = 0.026; RVD index: ARDS 1: 3.1 (3.0–4.2) vs. 4.6 (4.3–6.3), p = 0.026) (Table 2, Figs 2 and 3). However, this effect had already disappeared four hours after ARDS (Tables 3 and 4, Figs 2 and 3). This was mainly due to the rapid increase of the GI index and the RVD index after ARDS induction in the LAV group, which was followed by a decrease of both indices in the later phase of the study. It must be noted, however, that no statistically difference was observable in both target parameters after Bonferroni correction. Additionally, the Horowitz index showed an obvious conspicuousness (Fig 1). Furthermore, after eight hours of ARDS, 100% of the LPS animals still demonstrated moderate ARDS (PaO2/FiO2 101–200 mmHg). In contrast, only 60% of the LAV animals demonstrated moderate ARDS. The LPS group showed a higher HR and lactate, as well as a lower total urine output at all times. Overall, PIP seemed to be significantly higher in the lavage based model during the study (Tables 2–4), whereas compliance differed only in the early phase of ARDS between both experimental groups (ARDS 1: 12.0 (9.2–13.2) vs. 8.7 (6.7–12.2), p = 0.038). In contrast, SPAP, RR and cardiac output showed no differences between the experimental groups during ARDS (Tables 2–4).

## Discussion

In this study, two models of experimental ARDS in pigs, namely the lavage and the endotoxin models, were analyzed and compared with regard to EIT-derived parameters. Both models showed reliable EIT measurements, but the influences on the EIT parameters were quite different pointing out that the underlying pathophysiology affects the findings. The key result was that the GI index, which represents the disorder in ventilation distribution, and the RVD index, which is a measure for inhomogeneity as well, were higher in the LAV group initially. However, this effect disappeared during the study. It is particularly noteworthy that after Bonferroni correction no statistical significance was demonstrated. The endotoxin model led to more stable ARDS as compared to the lavage model, and no impact on the EIT-derived indices was observed in the LPS group. The well-established LAV model is frequently used to examine ventilation strategies in ARDS, but this study demonstrates that GI index and RVD index may be error-prone in the LAV model.

ARDS pathogenesis and pathophysiology is complex [20]. In patients, sepsis and pneumonia are the most common factor for the development of an acute lung injury [21], which can be imitated in animal studies by the intravenous injection or intratracheal application of LPS. Here, the mechanism of lung injury takes place in two ways: First, a direct effect leading to damage of the endothelial cells and second, a systemic inflammatory response. The endotoxin model is accompanied by hemodynamic instability [5,22,23].

In contrast, the well-established lavage model is characterized by surfactant depletion, which leads to an increase in alveolar surface tension. As a result, atelectasis and edema frequently occur, leading to impaired gas exchange [5,24]. The pathophysiological changes of this ARDS model also demonstrate some similarities with human ARDS, but the model fails to reproduce human ARDS completely. In particular, the lavage model is not able to mimic the inflammatory aspect and severe epithelial injury of human ARDS. Additionally, this model tends to instability with consecutive improved gas exchange [5,7]. Furthermore, saline solution may remain in the lungs after repetitive lavages. Nevertheless, the lavage model is frequently used for investigating ventilation strategies in ARDS, often in combination with EIT.

EIT is a bedside-available tool that enables continuous monitoring and evaluation of ventilation. Because of a huge amount of complex data, EIT-derived indices were introduced to analyze, structure and interpret these data. Two indices, which were studied in various experimental and clinical settings, are the GI index and the RVD index. The GI index was introduced by Zhao et al. to quantify tidal distribution [12]. Initially, Zhao et al. were able to demonstrate that the GI index may be useful to guide PEEP settings [25]. The idea of PEEP guidance by GI index was taken up in experimental and clinical studies several times [26,27]. Overall, the GI index focuses on spatial heterogeneity within the lung. Consequently, this parameter is a measure for ventilation inhomogeneity [12,28]. For that reason, it is used for evaluating ventilation strategies. However, we clearly demonstrated that GI index and RVD index were significantly initially higher in the LAV group but not in the LPS group as compared to the CO group. This effect disappeared during the study. It thus follows that the GI index and the RVD index were affected by the lavage model itself. Additionally, both indices were higher in the LAV group compared to the LPS group at the beginning, which may be the result of alveolar collapse and edema after the lavage, as well as remaining saline solution in the lung. This effect disappeared after a few hours as well. It should be noted that these findings could not be demonstrated fully after Bonferroni correction. In contrast, the GI index and the RVD index of the LPS group resembled that of the control group. This may be due to the fact that LPS induced lung injury is based on ubiquitous inflammatory damage of the endothelial cells, which is not initially accompanied by a high degree of inhomogeneity. Actually, the RVD index was developed to estimate homogeneity of the lung during slow inflation maneuvers [15]. Additionally, lung injury induced by LPS was more stable, which is presented in close interquartile ranges (Fig 1). More precisely, all six animals (100%) of the LPS group that finished the study protocol still demonstrated moderate ARDS eight hours after ARDS induction. In the LAV group, only 60% of the animals demonstrated moderate ARDS after eight hours. Furthermore, all animals in the LPS group needed catecholamine therapy due to hemodynamic instability on the basis of decreased blood pressure. In the LAV group, only one animal obtained catecholamine therapy to maintain blood pressure. But, this was one of the animals which died because of severe impairment of gas exchange. Additionally, volume therapy was more differentiated in the LPS group, although only significantly compared to CO group in the later phase of ARDS. Furthermore, total urinary output was less as well as HR and lactate were higher in the LPS group. This underlines the fact that an LPS based lung injury results in a septic clinical picture. Cardiac output did not differ between groups, but in both ARDS models, all considered respiratory mechanics changed significantly compared to the CO group.

Our study had some limitations to be addressed. First, the sample size of this study is low. Further studies must be performed to confirm these findings. This fact was underlined by the results after Bonferroni correction. Here, the significances could not be confirmed fully. Second, the RVD index was developed for slow inflation maneuvers. In this study, normal ventilation patterns were applied and even high respiratory rates with short inspiration times in the LPS and the LAV group during ARDS. The combination of short inspiration time and the few EIT sampling points introduce variations for individual animals. Finally, due to the position of the EIT belt at the 5^th^ intercostal space, ventilation distribution was visualized only at this section. However, the 5^th^ intercostal space seems to be one of the best electrode planes for estimating lung parameters [29].

## Conclusions

In summary, we examined if EIT parameters were influenced by different ARDS models, namely the LAV and the LPS models. We could clearly demonstrate that both models influenced the EIT measurements differently: whereas the lavage model demonstrated a related effect on the GI and RVD index, the LPS model did not reflect these findings. This implies that EIT parameters must be interpreted with regard to the chosen ARDS model. At the same time, this needs to be given careful consideration, especially when results are transferred. Therefore, EIT-guided human therapy might be limited. Additionally, the endotoxin induced ARDS model proved to be considerably more stable than the lavage based model. For those reasons, the selection of an adequate animal model is crucial. Even diagnostic, respectively monitoring parameters may be influenced by the model itself.

## Supporting information

S1 Table Supporting data set. (SAV)
